# HBOC-301 in Porcine Kidney Normothermic Machine Perfusion and the Effect of Vitamin C on Methemoglobin Formation

**DOI:** 10.3390/antiox11071329

**Published:** 2022-07-06

**Authors:** Eileen Edgworth, Lisa Ernst, Zoltan Czigany, Turgay Saritas, Laura Sophie Zarnitz, Marc Wiartalla, Peter Boor, Eva Miriam Buhl, Rolf Rossaint, René H. Tolba, Benedict Doorschodt, Gregor Fabry, Christian Bleilevens

**Affiliations:** 1 Department of Anesthesiology, Medical Faculty, University Hospital RWTH Aachen, 52074 Aachen, Germany; eedgworth@ukaachen.de (E.E.); lauzarnitz@ukaachen.de (L.S.Z.); rrossaint@ukaachen.de (R.R.); gregorfabry@hotmail.com (G.F.); 2 Institute for Laboratory Animal Science and Experimental Surgery, Faculty of Medicine, RWTH Aachen University, 52074 Aachen, Germany; lernst@ukaachen.de (L.E.); rtolba@ukaachen.de (R.H.T.); bdoorschodt@ukaachen.de (B.D.); 3 Department of Surgery and Transplantation, University Hospital RWTH Aachen, 52074 Aachen, Germany; zczigany@ukaachen.de; 4 Division of Nephrology and Immunology, University Hospital RWTH Aachen, 52074 Aachen, Germany; tsaritas@ukaachen.de; 5 Institute of Computer Sciences 11, RWTH Aachen University, 52074 Aachen, Germany; wiartalla@embedded.rwth-aachen.de; 6 Institute of Pathology, Division of Nephrology, Medical Faculty, RWTH Aachen, 52074 Aachen, Germany; pboor@ukaachen.de (P.B.); ebuhl@ukaachen.de (E.M.B.)

**Keywords:** normothermic machine perfusion, kidney preservation, kidney transplantation, ischemia reperfusion injury, hemoglobin-based oxygen carrier

## Abstract

Normothermic machine perfusion (NMP) of kidneys in combination with an optimized perfusate composition may increase donor organ preservation quality, especially in the case of marginal donor grafts. Optimization of currently employed perfusates is still a subject of present research. Due to the advantages of being cell-free, easy to store, and having minimal antigenicity, hemoglobin-based oxygen carriers, such as HBOC-301 (Oxyglobin^®^, Hemoglobin Oxygen Therapeutics LLC, Souderton, PA, USA), offer an alternative to the commonly used perfusates based on packed red blood cells (pRBC). As previously described, using HBOC results in formation of methemoglobin (metHb) as an adverse effect, inducing hypoxic conditions during the perfusion. As a potential counterpart to metHb formation, the application of the antioxidant ascorbic acid (VitC) is of high interest. Therefore, this study was conducted in four experimental groups, to compare the effect of NMP with (1) HBOC or (3) pRBC, and additionally examine a beneficial effect of VitC in both groups (2) HBOC + VitC and (4) pRBC + VitC. All groups were subjected to NMP for 6 h at a pressure of 75 mmHg. Kidneys in the HBOC groups had a significantly lower renal blood flow and increasing intrarenal resistance, with reduced renal function in comparison to the pRBC groups, as demonstrated by significantly lower creatinine clearance and higher fractional sodium excretion rates. Clinical chemistry markers for tissue damage (LDH, lactate) were higher in the HBOC groups, whereas no significant histological differences were observed. Although the application of VitC decreased oxidative stress levels, it was not able to significantly increase the outcome parameters mentioned above in either group. This study demonstrated that HBOC-301 is inferior to pRBCs in our porcine kidney NMP model, independent of additional VitC administration. Oxidative stress and fragmentation of the hemoglobin polymers could be detected as a possible reason for these results, hence further research, focusing on the use of cell-free oxygen carriers that do not exhibit this complex of issues, is required.

## 1. Introduction

Normothermic machine perfusion (NMP) is a promising experimental approach for preservation, as well as assessment and treatment, of kidney transplant grafts to improve organ quality and, ultimately, clinical outcomes [1]. This applies especially for donation after cardiac death (DCD) and donation from extended-criteria donors (ECD) [1,2]. In the majority of ex vivo perfusion studies, red blood cells were used as oxygen carriers in the perfusion solutions either as packed red blood cells (pRBCs) or whole blood including white blood cells (WBC) and platelets (PLT), or other variations [2]. However, pRBCs have several limitations regarding their use in NMP.

During NMP, erythrocytes are exposed to mechanical stress by the blood pump, sheer stress, and contact with the artificial surface of the perfusion system, which consists, at least, of gold standard-biocompatible components derived from cardiopulmonary bypass and extracorporeal membrane oxygenation (ECMO) systems. Clinical studies indicate hemolysis and subsequent increase of serum-free hemoglobin as common side-effects when using ECMO systems [3]. This problem might be potentially aggravated with increased duration of machine perfusion. Serum-free hemoglobin was shown to be of substantial importance for clinical outcomes. Hokka et al. found an association with postoperative kidney injury [4], and Omar et al. established it as an independent predictor for mortality in ECMO patients [5].

Additionally, a recent publication by Callaghan et al. expressed concerns about the underappreciated complexities of the use of third-party pRBCs in NMP. It listed, among others, AB0 and minor blood group compatibility, necessary crossmatching to donor or recipient, and risks of infection, as well as administrative issues as possibly relevant factors, because, even after perfusate washout, low quantities of pRBCs remain in the grafts [6].

Furthermore, pRBC availability is dependent on donors. pRBCs have to be refrigerated and storage life is limited to a maximum of 42 days with older pRBCs being associated with hemolysis and potentially poorer outcome, especially in patients with acute kidney injury [7,8].

These shortcomings of pRBCs have instigated the use of alternative, cell-free, oxygen-carriers. Hemoglobin-based oxygen carrier-201 (HBOC-201) (Hemopure, Hemoglobin Oxygen Therapeutics LLC, Souderton, PA, USA) is an experimental alternative oxygen-carrier and consists of glutaraldehyde-polymerized bovine hemoglobin with an average molecular weight of 250 kDa. It has been used in several different kidney machine perfusion studies [9,10,11]. In comparison to NMP with pRBCs, NMP using HBOC-201 showed equivalent results for most parameters as well as histological damage [11].

The main disadvantage of using HBOC-201 for kidney perfusion is that it has only been approved by regulatory authorities in South Africa and Russia and is still experimental in other countries. In contrast, HBOC-301 (Oxyglobin, Hemoglobin Oxygen Therapeutics LLC, Souderton, PA, USA) is approved for veterinary use and therefore features a better availability for preclinical studies. It also consists of glutaraldehyde-polymerized bovine hemoglobin, although with a lower average molecular weight of 200 kDa.

A known problem of glutaraldehyde-polymerized bovine hemoglobin is formation of methemoglobin (metHb) by oxidation of the ferrous (Fe^2+^) iron ions to the ferric (Fe^3+^) state, which does not bind oxygen. A study investigating gradual kidney rewarming also used HBOC-201 in NMP for simulated reperfusion and reported metHb levels of approximately 20% after 120 min of NMP [10], while in other studies using HBOC-201 in normothermic or subnormothermic machine perfusion, metHb levels were not reported [9,11]. Besides methylene blue, more common clinically applied ascorbic acid can be used to mitigate methemoglobinemia due to its reducing and radical scavenging potential [12]. A study by Cooper et al. suggests VitC’s efficacy in reducing methemoglobinemia associated with the application of HBOCs [13] and its safe, beneficial utilization in NMP has already been established by our working group [14]. However, so far no studies exploring the effect of VitC on HBOC in a NMP-setting exist.

In this study, we evaluated kidney function and injury during NMP using HBOC-301 in comparison to a pRBC-based perfusate, both with and without addition of VitC to reduce methemoglobin formation and oxidative stress. For this purpose we analyzed functional parameters, such as renal blood flow, urine production and intrarenal resistance (IRR), and sodium, potassium, and creatinine levels in both urine and blood samples to estimate fractional sodium and potassium excretion as well as creatinine clearance, and lactate and LDH were assessed as markers for kidney damage. To our knowledge, this is the first study using HBOC-301 in porcine kidney NMP.

## 2. Materials and Methods

The experimental protocol was approved by the Institutional Animal Care and Use Committee of the RWTH Aachen University Hospital and performed in accordance with German legislation governing animal studies following the ‘Guide for the Care and Use of Laboratory Animals (NIH publication, 8th edition, 2011) and the Directive 2010/63/EU on the protection of animals used for scientific purposes (Official Journal of the European Union, 2010).

### 2.1. Preparation of pRBCs

Female German landrace pigs weighing 45–55 kg were premedicated with 8 mg/kg BW azaperone (Stresnil, Janssen-Cilag GmbH, Neuss, Germany), 15 mg/kg BW ketamine (Ceva GmbH, Duesseldorf, Germany), and 10 mg atropine (1 mL/1% atropine sulfate, Dr. Franz Köhler Chemie GmbH, Bensheim, Germany) administrated intramuscularly. Animals were sacrificed by overdose of pentobarbital followed by subsequent exsanguination by cannulation of the femoral vein. On average, 1800 mL blood could be obtained, collected into sodium-citrate filled blood bags (Composelect^®^, Fresenius Kabi AG, 61346 Bad Homburg, Germany). Blood separation was achieved through sedimentation by placing each bag at a 45° inclination for a minimum of 3 h, after which the cell layer was transferred into another blood bag filled with SAG-M solution and depleted of leukocytes. This method of preparation was investigated by Gifford et al. [15] and the quality and durability of the thus-acquired porcine pRBCs in our setting were tested by our working group ahead of this study. The finalized pRBCs were stored at a temperature of 4 °C for 2 to 5 days for subsequent experiments.

### 2.2. Kidney Retrieval and Cannulation

**Figure 1:**
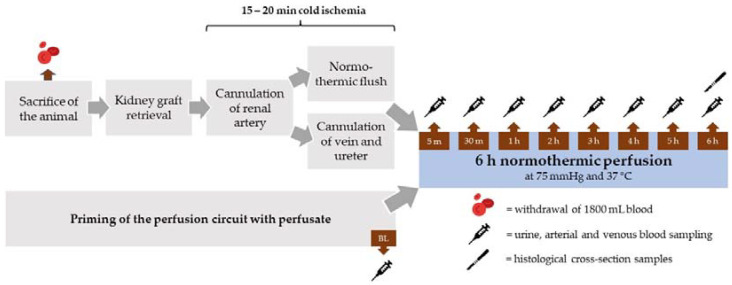
Schematic overview of the experimental procedure.

After sacrifice of the animals, both kidneys were retrieved within a 10 to 15 min time period. The kidneys were assigned randomly to the different perfusate groups (*n* = 5 per group). The renal artery was cannulated and the organ flushed with 500 mL heparinized (5000 IU/mL) Ringer’s solution at 37 °C at pressure of 75 cm H_2_O. In parallel, the renal vein and ureter were cannulated (Figure 1).

### 2.3. Perfusate

The perfusion circuit was prefilled with 250 mL of perfusion buffer supplemented with either 250 mL HBOC-301 (groups 1 and 2) or 250 mL of the prepared porcine pRBCs (groups 3 and 4) (Table 1). The perfusion buffer consisted of 5 g mannitol, 2.1 g sodium bicarbonate, 1.98 g glucose, and 0.116 g creatinine per liter of Ringer’s solution. Then, 5000 UE of heparin (LEO Pharma GmbH, Neu-Isenburg, Germany) was added to the systems in all groups.

In groups 1 and 3, ascorbic acid (vitamin C, ROTEXMEDICA GmbH, Trittau, Germany) was added as a bolus at an amount of 330 µmol after the start of perfusion as well as continuously at a rate of 330 µmol/h during the entire perfusion period (Table 1). This amount was chosen due to pre-testing of the effect of varying doses of VitC on methemoglobinemia in HBOC-301 in a mock-loop setting and in consideration of the dose previously used by our working group in a study with pRBC [14].

During NMP, 11.2 mL/h of a nutrient solution comprised of Nutriflex^®^ peri (B. Braun, Melsungen, Germany), Cernevit (Baxter, Unterschleißheim, Germany) and insulin (Insuman Rapid, Sanofi-Aventi, Frankfurt a.M., Germany) was added to all groups, as previously described by our group [16].

### 2.4. Normothermic Machine Perfusion System

The NMP system consisted of a 1 L hard shell reservoir (Terumo Cardiovascular Systems, Leuven, Belgium), a centrifugal blood pump (Affinity™ CP, Medtronic Inc., Minneapolis, MI, USA) that was controlled via a custom-made pump-drive, and a newborn oxygenator (Eurosets S.r.l., Medolla, Italy).

The pressure in the renal artery was monitored through an in-line pressure sensor in the arterial cannula connected to a customary patient monitor (Philips Medizin Systeme Boeblingen GmbH, Boeblingen, Germany), which was backcoupled to the pump-drive, enabling constant and automatic mean perfusion pressure control. The flow was measured using a flow computer with a clamp-on transducer (Flowmeter Sono-TT Ultrasonic, em-tec, Finning, Germany) and a constant perfusate temperature of 37 °C was maintained through a water bath (DC3, Thermo Haake, Karlsruhe, Germany) connected to the oxygenator. At the start of perfusion, the pressure was continually increased over the course of 5 min from 25 mmHg to 75 mmHg, which was previously described as a well-suited pressure for kidney NMP settings [17,18]. This perfusion pressure was automatically maintained via the custom-made pump drive for the subsequent 6 h of the experiment. Fluid loss due to urine production was compensated by providing Ringer’s solution to the perfusate through a custom-made automated system consisting of a level sensor for the hard shell reservoir and a roller pump.

### 2.5. Sampling and Laboratory Analysis

Samples of arterial and venous perfusate and urine were collected at nine sampling points (baseline, 5 min, 30 min, 60 min, 120 min, 180 min, 240 min, 300 min, and 360 min perfusion) and the total volume of the secreted urine was measured. Blood-gas analysis (ABL 800 flex, Radiometer GmbH, Krefeld, Germany) was performed on all samples. After centrifugation, the supernatants of the arterial samples as well as urine samples were stored at −80 °C for later laboratory and biochemical analyses.

Oxidative stress in perfusate samples was assessed by measuring the static oxidation reduction potential (sORP) and the antioxidant capacity using RedoxSYS Diagnostic SystemTM (Aytu BioScience, Inc., Englewood, CO, USA), as previously described by our working group [14]. This method was also shown to adequately quantify levels of oxidative stress in clinical studies carried out by Panigrahi et al. [19] and Rael et al. [20]. Perfusate samples were additionally analyzed for creatinine, iron, urea, aspartate aminotransferase (AST), lactate dehydrogenase (LDH), and total protein by the local ISO 9001:2015-certified laboratory at the Institute for Laboratory Animal Science and Experimental Surgery, while the urine samples were analyzed for creatinine, urea, and urine protein. Creatinine clearance rate (CrCl, urinary creatinine ∗ urinary flow/serum creatinine), intrarenal resistance (arterial pressure/flow), fractional potassium excretion (urinary potassium ∗ serum creatinine/serum potassium ∗ urinary creatinine), and fractional sodium excretion (urinary sodium ∗ serum creatinine/serum sodium ∗ urinary creatinine) were calculated.

### 2.6. Histology

After 6 h perfusion, histological cross-section samples were taken from the medulla and cortex of each kidney and fixated in 4% formaldehyde. Tissue samples were processed, hematoxylin-eosin (HE) stained and subsequently assessed by an expert in pathology and experimental nephrology by using a scoring system for tubule injury, hypokalemic nephropathy, and osmotic nephropathy (0 = no; 1 = mild; 2 = moderate; 3 = severe injury) [14]. These scoring systems have been established in previous studies assessing kidney damage in a transplant setting [21,22,23].

### 2.7. Electron Microscopy

For electron microscopy, small tissue pieces were fixed in 3% glutaraldehyde in 0.1 M Sorensen’s phosphate buffer (PBS). Then, samples were washed in PBS, post-fixed in 1% OsO4 (Roth, Karlsruhe, Germany) in 25 mM sucrose buffer (Merck, Darmstadt, Germany), and dehydrated by ascending ethanol series (30%, 50%, 70%, 90%, and 100%) for 10 min each. The last step was repeated three times. Dehydrated specimens were incubated in propylene oxide (Serva, Heidelberg, Germany) for 30 min, in a mixture of Epon resin (Serva, Heidelberg, Germany) and propylene oxide (1:1) for 1 h, and finally in pure Epon for 1 h. Samples were embedded in pure Epon. Epon polymerization was performed at 90 °C for 2 h. Ultrathin sections (70–100 nm) were stained with 0.5% uranyl acetate and 1% lead citrate (both EMS, Munich, Germany) to enhance the contrast. Samples were viewed at an acceleration voltage of 60 kV using a Zeiss Leo 906 (Carl Zeiss, Oberkochen, Germany) transmission electron microscope.

### 2.8. Statistical Analysis

Statistical analysis was carried out using GraphPad Prism 9.2.0 software package (GraphPad Software Inc., San Diego, CA, USA). Mean and standard error of the mean (SEM) were calculated for each point of time and outliers were eliminated using the Grubb’s test. A multiple comparison followed by a Šidák post-test correction was performed comparing all four groups for all measurements during perfusion. For the comparison of histological damage scores between the four groups, one-way ANOVA was applied and for hypokalemic nephropathy the chi-square test was executed. Data are presented as mean ± SEM and a *p*-value < 0.05 was considered statistically significant.

## 3. Results

### 3.1. Kidney Weights

Kidneys were weighed after retrieval from the animal and again after 6 h perfusion. Kidney weights before perfusion did not differ between the four groups (119 ± 20.1 vs. 111.2 ± 9.0 vs. 118.4 ± 11.8 vs. 118.4 ± 13.9; HBOC + VitC vs. HBOC vs. pRBC + VitC vs. pRBC, respectively). While in all groups weight gains could be detected (+71.2 ± 20.2 vs. +100.6 ± 26.4 vs. +99.0 ± 20.5 vs. +83.8 ± 19.7; HBOC + VitC vs. HBOC vs. pRBC + VitC vs. pRBC, respectively), no significant differences in weight gain could be observed due to strong variations within the respective groups. The average warm ischemic time was ≤ 30 min in all groups (29.2 ± 8.6 vs. 24.4 ± 4.0 vs. 26.2 ± 4.3 vs. 30.0 ± 4.7; HBOC + VitC vs. HBOC vs. pRBC + VitC vs. pRBC, respectively).

### 3.2. Perfusion Parameters

**Figure 2:**
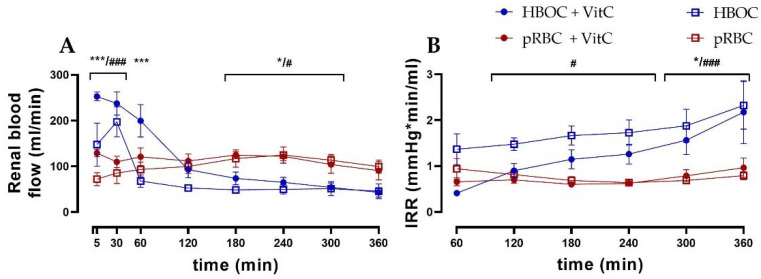
(**A**) Renal blood flow in the HBOC groups was initially higher than in the pRBC groups; however, it decreased and remained significantly lower between 180 min and 300 min perfusion. (**B**) Intrarenal resistance in the HBOC group was significantly higher than in the pRBC groups from 120 min onwards and in the HBOC + VitC group from 300 min (HBOC + VitC vs. pRBC + VitC; * *p* < 0.05, *** *p* < 0.001, HBOC vs. pRBC; # *p* < 0.05, ### *p* < 0.001).

Renal blood flow differed significantly between the HBOC and the pRBC groups (Figure 2A). While the HBOC groups showed a characteristic peak in blood flow during the first 60 min, which then declined rapidly below the other groups’ levels, the pRBC groups maintained a constant blood flow throughout the 6 h perfusion. The intrarenal resistance (IRR) remained stable over time in the pRBC groups, while IRR increased in the HBOC groups after 120 min of perfusion (Figure 2B).

### 3.3. Renal Function

**Figure 3:**
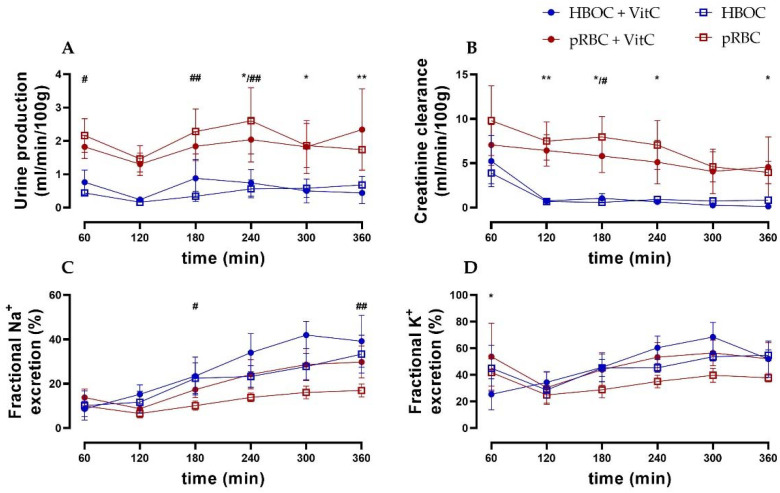
(**A**) Urine production was higher in both pRBC groups compared to the HBOC groups. (**B**) Creatinine clearance in the HBOC groups decreased considerably after 120 min, while the pRBC groups maintained a significantly higher level. (**C**) Fractional sodium and (**D**) fractional potassium excretion showed a tendency towards lower salt excretion in the pRBC than in the HBOC groups; * *p* < 0.05, ** *p* < 0.01 (HBOC + VitC vs. pRBC + VitC), # *p* < 0.05, ## *p* < 0.01 (HBOC vs. pRBC).

Urine production remained constant in all groups. However, it was higher in the pRBC groups compared to the HBOC groups with significant differences at 180 min and 240 min (pRBC vs. HBOC) and at 240, 300, and 360 min (pRBC + VitC vs. HBOC + VitC) (Figure 3A).

Creatinine clearance (CrCl) decreased over time in all groups, with the pRBC groups showing a trend of preserving higher CrCl compared to the HBOC groups, reaching significance at 180 min (pRBC) and at 120 min, 180 min, 240 min, and 360 min perfusion (pRBC + VitC) (Figure 3B). Fractional sodium and potassium excretion (marker of impaired tubule salt reabsorption) increased over time in all groups, with the pRBC groups showing a tendency towards maintaining lower salt excretion without reaching significance at most time points (Figure 2D and Figure 3C).

### 3.4. Blood-Gas Analysis

**Figure 4:**
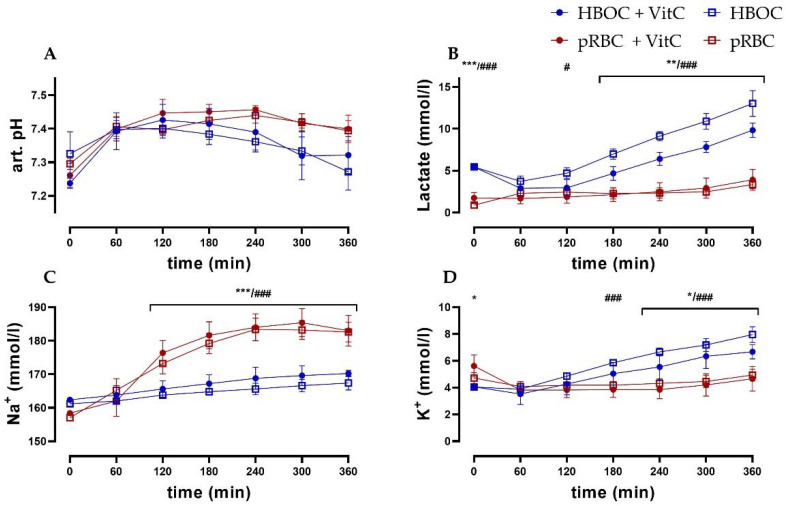
(**A**) Arterial pH, (**B**) lactate, and (**C**) perfusate sodium concentrations were significantly higher in the pRBC groups vs. the HBOC groups from 120 min onwards and (**D**) potassium concentrations remained significantly lower after 180 min (pRBC vs. HBOC; # p < 0.05, ### *p* < 0.001) and 240 min (pRBC + VitC vs. HBOC + VitC; * *p* < 0.05, ** *p* < 0.01, *** *p* < 0.001).

Arterial pH levels remained comparable between the HBOC and the pRBC groups. While in the pRBC groups, pH levels were more stable, significant differences between the groups could not be observed (Figure 4A). Lactate levels in the pRBC groups were constant over the perfusion period, while they increased in the HBOC groups, reaching significant difference after 120 min (pRBC + VitC vs. HBO + VitC) and 180 min (pRBC vs. HBOC) (Figure 4B). Perfusate sodium concentrations increased significantly in the pRBC groups, whereas in the HBOC groups potassium concentration increased, reaching significance after 180 min (pRBC vs. HBOC) and at 240 min (pRBC + VitC vs. HBO + VitC) (Figure 4C,D).

### 3.5. Kidney Damage

**Figure 5:**
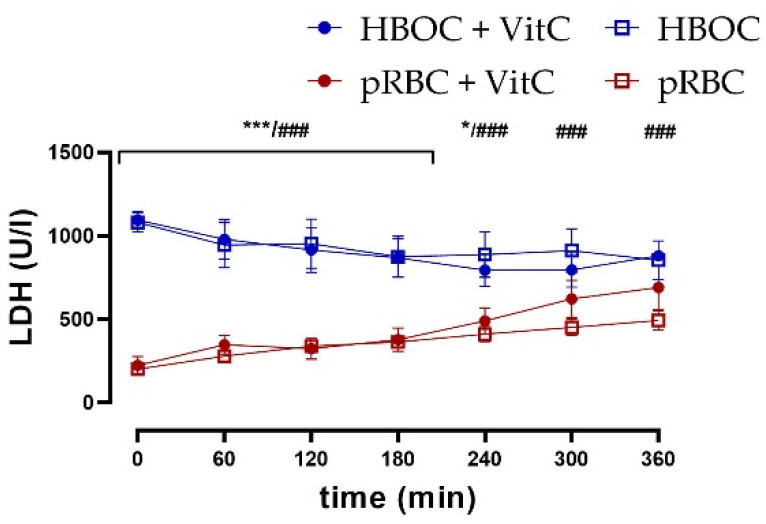
LDH perfusate concentrations were significantly higher in the HBOC groups than in pRBC groups without VitC throughout 6 h perfusion (HBOC vs. pRBC; ### *p* < 0.001) and until 240 min with VitC (HBOC + VitC vs. pRBC + VitC 0–180 min; *** *p* < 0.001, 240 min * *p* < 0.05).

Kidney tissue damage was assessed via measurement of lactate dehydrogenase (LDH) in the perfusate. LDH was significantly lower in the pRBC than in HBOC groups without VitC throughout the perfusion time and until 240 min with VitC, whereas the addition of VitC could not decrease levels and thus showed no protective effects (Figure 5).

### 3.6. Oxidative Stress

**Figure 6:**
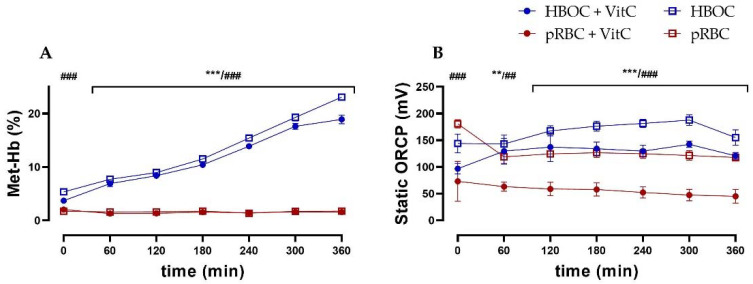
(**A**) Significantly higher levels of methemoglobin in the HBOC groups during 6 h perfusion without VitC and after 60 min with VitC. and (**B**) static oxidation reduction potential (ORCP) (HBOC + VitC vs. pRBC + VitC; ** *p* < 0.01, *** *p* < 0.001, HBOC vs. pRBC; ## *p* < 0.01, ### *p* < 0.001).

In the HBOC group, a constant methemoglobin formation up to a concentration of 23.1 ± 1.34% of total hemoglobin after 6 h of perfusion could be observed. This increase could only be partially mitigated by the administration of the antioxidant vitamin C, with the HBOC + VitC group reaching a significantly lower methemoglobin concentration of 18.9 ± 1.63% after 6 h. In the pRBC groups, methemoglobin formation did not occur and concentrations stayed at a physiological level of <2% at all times (Figure 6A).

As an indicator of oxidative stress, static oxidation reduction potential (ORCP) was higher in both HBOC groups than the respective pRBC groups (without VitC from 0 min, with VitC from 60 min onwards). Between the pRBC groups, vitamin C was able to significantly reduce oxidative stress during the entire perfusion time (Figure 6B), whereas in the HBOC groups, only a tendency could be shown.

### 3.7. Hemoglobin Fragmentation

**Figure 7:**
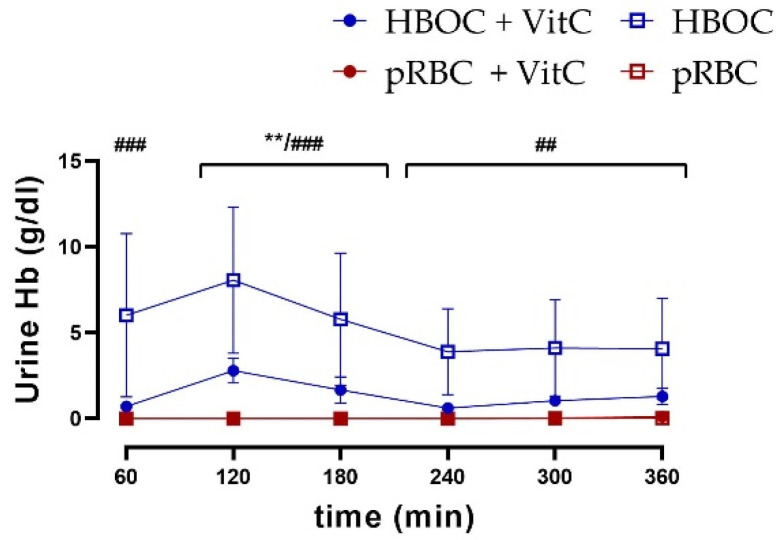
Hemoglobin concentration in urine was significantly higher in the HBOC group than in the pRBC and pRBC + VitC groups (HBOC + VitC vs. pRBC + VitC; ** *p* < 0.01, HBOC vs. pRBC; ## *p* < 0.01, ### *p* < 0.001).

In comparison to the pRBC groups, a higher urine excretion of hemoglobin was seen in both HBOC groups. Especially in the groups without the antioxidant effect of vitamin C, urine hemoglobin levels remained significantly higher than in the pRBC groups, with a peak of 8.06 ± 9.52 g/dL (Figure 7).

### 3.8. Histological Analysis

Microscopic assessment of HE-stained kidney cross-sections after 6 h perfusion revealed signs of tubular injury, osmotic nephropathy, and hypokalemic nephropathy in all four groups. The mean tubular injury severity score in both the pRBC groups was 2.1 ± 0.224 with the HBOC group scoring at 1.9 ± 0.652 and 1.7 ± 0.274 for HBOC + VitC on a scale of 0 to 3 and no significant differences between either of the groups.

**Figure 8:**
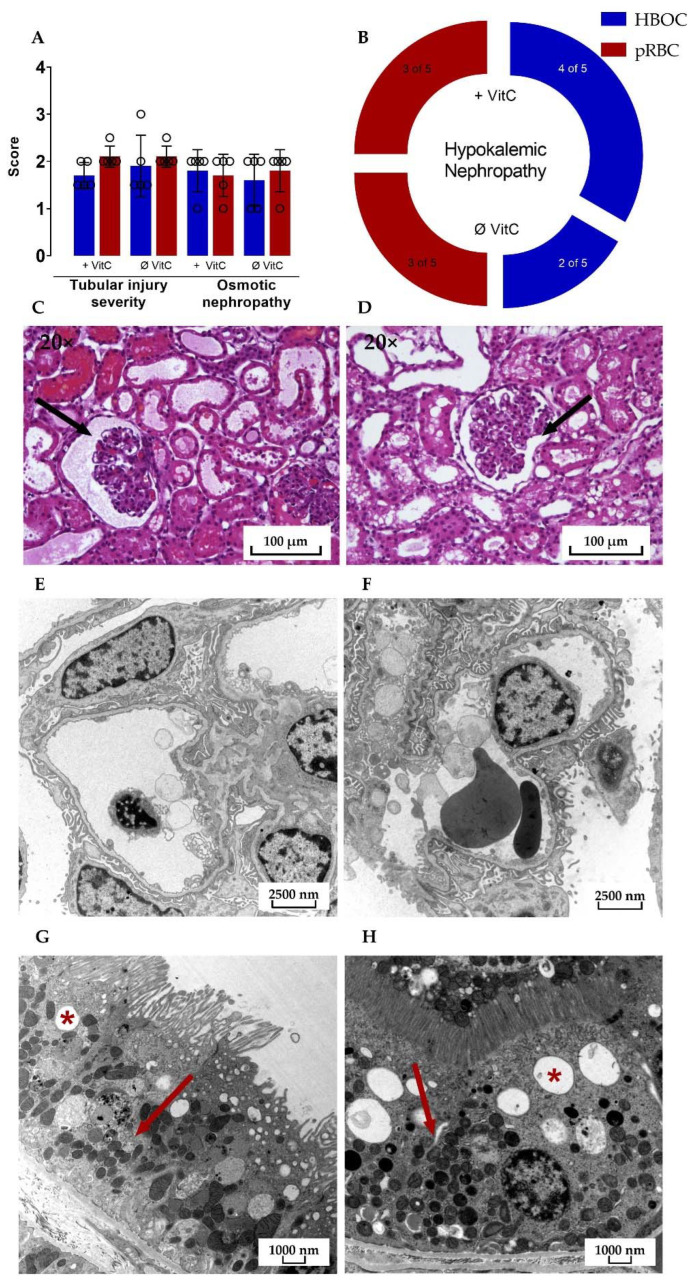
(**A**) Significant differences between the HBOC and the pRBC groups were not seen in the histological examination for tubular injury and osmotic nephropathy and (**B**) hypokalemic nephropathy. Signs of tubular damage and discreet dilation of the Bowman capsule (➔) were present in all four groups; examples of both HBOC- (**C**) and pRBC-group kidneys (**D**) are shown (hematoxylin–eosin stained, 20× magnified). In transmission electron microscopy, intact glomeruli (**E**,**F**) and proximal tubular cells with only scarce signs of damage (*: examples of vacuolization; ➔: intact mitochondria) could be detected in all four groups; examples of both HBOC (**E**,**G**, 4646× magnified) and pRBC group kidneys (**F**,**H**, 6000× magnified) are shown.

Transmission electron microscopy showed similar results in HBOC and pRBC groups, with intact glomerular filtration barriers and densely interdigitating podocyte pedicels in all groups (Figure 8E,F). Proximal tubules were also mostly without damage, with only a small amount of cells in all four groups presenting vacuolization and signs of mitochondrial damage, such as rarefication of cristae.

## 4. Discussion

The aim of this study was to investigate whether, for future clinical application, the oxygen-carrier HBOC-301, with potential key advantages over other, cell-based, oxygen carriers, could be an equivalent or even superior alternative to the use of pRBCs in a 6 h normothermic kidney perfusion setting, particularly regarding functional outcome and kidney damage. Furthermore, we aimed to assess whether administration of vitamin C could prevent potentially deleterious methemoglobin formation and thus facilitate the usage of HBOC-301 for normothermic kidney perfusion.

The main outcome of the study was that kidneys perfused with HBOC-301 showed a significantly lower renal blood flow and urine production in comparison to pRBC-perfused kidneys. They also exhibited a continuously increasing intrarenal resistance over the course of perfusion, suggesting a lower functional level, accompanied by a lower renal clearance. Regardless of its antioxidant potential, which we were able to display through measurement of static oxidation reduction potential, VitC could not significantly improve the functional parameters mentioned above in either the HBOC or pRBC groups. Overall kidney damage appeared to be more severe in the HBOC groups as indicated by higher levels of LDH and lactate, although this could not be confirmed by histological assessment. For most parameters, the differences between HBOC and pRBC groups were most prominent after approximately 120 min to 180 min.

The utilization of cell-free oxygen carriers in ex-vivo kidney perfusion models has previously been investigated by different groups, with generally positive outcomes. The functionally related HBOC-201 compound was examined in a subnormothermic machine perfusion model of porcine kidneys by Bhattacharjee et al. [11] and in a normothermic perfusion model on discarded human kidneys by Aburawi et al. [9], with both studies presenting functional and histological outcomes comparable to the pRBC groups.

The different outcome in our study could be linked to the different perfusate we used, consisting of HBOC without blood in contrast to Bhattacharjee et al. perfusing with a mixture of HBOC and blood [11]. Furthermore, in our study HBOC-301 was used instead of HBOC-201 because of its better availability. While HBOC-201 is only medically approved in South Africa, HBOC-301 holds a pre-existing veterinary admission in the EU and offers the same advantages of being a cell-free oxygen carrier based on polymerized hemoglobin. The major difference between the two HBOCs, relevant for kidney perfusion, is the difference in tetrameric hemoglobin content. Although both HBOCs are comprised primarily of polymerized Hb (MW > 65 kDa) and stabilized Hb tetramer and dimer components each comprises 1.5% of all Hb in HBOC-201. By comparison, Hb tetramer and dimer components comprise 30% and 3.5%, respectively, of all Hb in HBOC-301 [24], which could lead to a quicker dissociation into dimers or hemoglobin subunits. This hypothesis is supported by the detection of considerable concentrations of urine hemoglobin in the HBOC groups, leading to the conclusion that there was renal filtration of hemoglobin in the HBOC-perfused kidneys. Since hemoglobin tetramers have a low sieving coefficient of about 0.03, and therefore physiologically only small amounts that can be resorbed by the kidney are filtrated, the massive urinal excretion of hemoglobin observed indicates the existence of smaller hemoglobin-subunits in the HBOC groups, such as dimers or monomers.

Free serum hemoglobin was previously described as having adverse effects on kidneys, with extravasated hemoglobin causing nephrotoxicity and scavenging of nitric oxide (NO), thus leading to hypertension and higher intrarenal resistance [25,26]. The latter could also be observed in the HBOC groups in our study, while a possible obstruction of glomeruli could not be shown upon histological analysis.

Additionally, free hemoglobin shows a stronger tendency towards oxidation of the bound iron from the ferrous (Fe^2+^) to the ferric (Fe^3+^) state [12]. Consequently, the generated methemoglobin is unable to transport oxygen, with methemoglobinemia > 10% causing decreased oxygenation and functional anemia [27], a concentration that was surpassed in our HBOC groups at approximately 180 min perfusion.

Combined with the increased oxidative potential and proinflammatory effects of free hemoglobin, particularly in the extravascular compartment, methemoglobin can cause significant tissue damage [28]. Since these issues were encountered in preceding experiments, we hypothesized that adding vitamin C, a potent antioxidant which was previously employed to reduce methemoglobinemia associated with HBOCs [29], could counter the oxidative effects observed while using HBOC-301 in an ECMO setting. For this purpose, a concentration established in preliminary experiments with Oxyglobin was used. Although the addition of vitamin C was able to decrease oxidative stress in the respective HBOC and pRBC groups, which is in line with our previous study on the application of vitamin C in kidney NMP [14], it only marginally diminished the adverse effects of methemoglobinemia and hemoglobin-polymer dissociation without reaching significance, exhibiting a beneficial effect on the parameters urine hemoglobin, intrarenal resistance, and renal blood flow.

Several limitations have to be considered when evaluating our study. First, it remains unclear whether the insufficient antioxidant effect of vitamin C was caused by underdosing. Preceding experiments to find an effective dose for the initial bolus as well as the continuous infusion rates were performed in a mock loop setting. While underdosing did not show a sufficient effect in reducing methemoglobin, overdosing led to harmful effects on HBOC-301, such as faster degradation of hemoglobin tetramers. However, the optimal dose during NMP might differ from the in vitro experiments, for example due to potential antioxidant capacities of renal cells.

Furthermore, in contrast to other groups, we used porcine kidneys instead of discarded human donor kidneys because of availability and legal implications. However, porcine kidney NMP is an established and commonly used model worldwide.

Additionally, our number of kidneys per group was limited (*n* = 5), so as to adhere to animal protection standards of using the minimal possible amount of laboratory animals, while still being able to obtain statistically significant results.

When assessing the study parameters, it is noticeable that there were no significant histological differences to be found between the four groups. This could be attributed to the low number of kidneys, the relatively short NMP time of 6 h, or the magnitude of pre-existing damage evoked by ischemia during retrieval and cannulation outweighing injury induced by NMP, since tubule damage and dilation of the Bowman capsules was seen in all kidneys.

## 5. Conclusions

A perfusate containing hemoglobin-based oxygen carrier HBOC-301 (Oxyglobin^®^), could not achieve results equivalent to a pRBC-based perfusate in a 6 h kidney NMP setting. While having indisputable practical advantages over the usage of pRBC, the functional efficacy of HBOC-301 did not suffice and increased oxidative stress might have provoked relevant deterioration of outcome parameters. Addition of the antioxidant vitamin C to the perfusate could not improve organ quality.

The ideal composition of NMP perfusates still remains a subject of future investigation. In particular, the further enhanced HBOC-201 (Hemopure^®^) variant, as well as alternative types of cell-free oxygen carriers such as M101, which was used in hypothermic machine perfusion studies by Kasil et al. [30], are candidates for further examination, in order to improve NMP perfusates, aiming to increase the availability of kidney grafts for transplantation, in particular with regards to extended-criteria donor kidneys.

## Figures and Tables

**Table 1:** Perfusate composition of the four perfusion groups.

**Group 1: HBOC + VitC**	**Group 3: pRBC + VitC**
250 mL HBOC-301 (Oxyglobin^®^)	250 mL pig pRBC
250 mL perfusion buffer	250 mL perfusion buffer
Initial bolus of 330 μmol ascorbic acid	Initial bolus of 330 μmol ascorbic acid
Infusion of 330 μmol ascorbic acid/h	Infusion of 330 μmol ascorbic acid/h
**Group 2: HBOC**	**Group 4: pRBC**
250 mL HBOC-301 (Oxyglobin^®^)	250 mL pig pRBC
250 mL perfusion buffer	250 mL perfusion buffer

## Data Availability

The datasets generated and/or analyzed during the current study are available from the corresponding author upon reasonable request.
